# Cross-Cancer Characterization of CAMK2A Reveals Its Favorable Prognostic Association and Neuronal Signature Enrichment in Glioma

**DOI:** 10.3390/biomedicines14102216

**Published:** 2026-09-30

**Authors:** Yimin Pan, Changwu Wu, Jun Tan, Fushu Luo, Ziqiang Wu

**Affiliations:** 1 Department of Intensive Care Medicine, Xiangya Hospital, Central South University, 87 Xiangya St., Changsha 410008, China; yimin.pan@csu.edu.cn; 2 National Clinical Research Center of Geriatric Disorders, Xiangya Hospital, Central South University, 87 Xiangya St., Changsha 410008, China; 3 Department of Neurosurgery, Xiangya Hospital, Central South University, 87 Xiangya St., Changsha 410008, China; 4 Department of Pathology, Xiangya Hospital, Central South University, 87 Xiangya St., Changsha 410008, China

**Keywords:** CAMK2A, glioma, neuron-like state, single-cell RNA sequencing, prognosis

## Abstract

**Background:** Calcium/calmodulin-dependent protein kinase II alpha (*CAMK2A*) is a neuron-enriched kinase that plays essential roles in synaptic signaling and neuronal differentiation. However, its clinical significance and biological relevance in glioma remain incompletely understood. This study aimed to systematically characterize *CAMK2A* across human cancers and investigate its prognostic significance and association with neuronal differentiation programs in glioma. **Methods:** An integrated multi-omics analysis was performed using pan-cancer transcriptomic, proteomic, genomic, DNA methylation, bulk RNA-sequencing, and single-cell RNA-sequencing datasets from TCGA, CPTAC, GTEx, CGGA, and other public resources. *CAMK2A* expression patterns, clinical associations, prognostic value, molecular characteristics, pathway activities, and cellular localization were systematically evaluated. Multiplex immunofluorescence staining of human glioma specimens was further performed to validate the protein-level expression patterns of CaMKII and the neuronal marker MAP2. **Results:** *CAMK2A* exhibited lineage-specific dysregulation across human cancers and was progressively downregulated during glioma progression. Higher *CAMK2A* expression was consistently associated with prolonged overall survival across multiple independent glioma cohorts and remained an independent favorable prognostic factor after adjustment for established clinicopathological and molecular variables. Functional enrichment analyses linked *CAMK2A* to neuronal differentiation, synaptic organization, calcium signaling, and neurotransmission-related pathways. Gliomas with high *CAMK2A* expression exhibited lower scores for several malignancy-associated pathways and enrichment of neuronal transcriptional signatures; these tissue-level associations do not distinguish tumor-intrinsic programs from normal neuronal admixture. Single-cell transcriptomic analyses further revealed preferential enrichment of *CAMK2A* in neuronal populations. Consistently, multiplex immunofluorescence demonstrated a coordinated reduction in CaMKII and MAP2 protein expression with glioma progression. **Conclusions:** *CAMK2A* represents a neuron-associated biomarker linked to preserved neuronal differentiation programs and favorable clinical outcomes in glioma. These findings support its potential value as a prognostic biomarker and provide further insight into the relationship between neuronal differentiation programs and glioma progression.

## 1. Introduction

Calcium/calmodulin-dependent protein kinase II (CaMKII) is one of the principal intracellular effectors of calcium signaling in the central nervous system (CNS), translating transient calcium/calmodulin signals into sustained cellular responses [1,2,3]. Through this mechanism, CaMKII regulates diverse neuronal processes, including neuronal differentiation, dendritic spine development, synaptic maturation, activity-dependent gene transcription, and learning and memory [1,2,3]. The CaMKII family comprises four isoforms, *CAMK2A, CAMK2B, CAMK2D*, and *CAMK2G*, among which *CAMK2A* is highly enriched in excitatory neurons and mature postsynaptic structures [2,4]. Owing to its remarkable neuronal specificity, *CAMK2A* is widely regarded as a representative molecular marker reflecting neuronal maturation and excitatory neuronal network activity.

At the synaptic level, CaMKII serves as a central regulator linking neuronal activity to synaptic remodeling [4]. Synaptic stimulation-induced calcium influx rapidly activates CaMKII, which subsequently regulates AMPA receptor trafficking, NMDA receptor-associated signaling, postsynaptic density assembly, actin cytoskeleton remodeling, and long-term potentiation [4,5,6]. Consequently, CaMKII not only mediates rapid modulation of synaptic transmission but also maintains dendritic spine architecture and the stability of postsynaptic protein complexes, thereby contributing to neuronal circuit remodeling during development, learning, and pathological conditions [2,4].

Notably, neuronal activity has emerged as an important regulator of neuroimmune homeostasis in the CNS [7,8]. As the fundamental structural unit for neuronal communication, the synapse serves not only as a platform for information transmission but also as a dynamic interface integrating neuronal activity, glial responses, and local immune signaling [9,10]. Aberrant or sustained neuronal activity reshapes the neuroimmune microenvironment by regulating microglial surveillance, complement-mediated synaptic pruning, and inflammatory cytokine release, collectively forming the biological basis of synaptic immunity and neuroimmune regulation [11,12]. As increasing evidence highlights the contribution of neuronal activity and neuroimmune signaling to diverse pathological conditions, CaMKII-mediated activity-dependent signaling has also been implicated in a broad spectrum of human diseases, including cancer [4,10,13].

Increasing evidence from multiple cancer types indicates that CaMKII participates in the regulation of tumor cell proliferation, apoptosis, invasion, epithelial–mesenchymal transition (EMT), and metabolic reprogramming [14]. Notably, the biological functions of CaMKII exhibit marked tumor-type specificity [15]. CaMKII predominantly exerts oncogenic functions in breast cancer and hepatocellular carcinoma by promoting tumor growth and metastasis [16,17], whereas it displays tumor-suppressive activities in several other malignancies, suggesting that its biological roles are profoundly influenced by tissue origin and microenvironmental context [15]. In contrast to peripheral solid tumors, tumors arising within the CNS possess a unique biological background in which *CAMK2A* exhibits highly restricted neuronal expression, raising the possibility that its functional roles in brain tumors may differ from those in non-neural malignancies [1,18]. Moreover, neuron–glioma interactions, neuron-like cellular states, and activity-dependent synaptic signaling have recently emerged as key mechanisms driving glioma heterogeneity, invasiveness, and disease progression [18,19]. Nevertheless, whether *CAMK2A* is associated with neuron-like differentiation programs and neuronal transcriptional signatures in glioma remains largely unknown, and its clinical significance and cellular localization have yet to be systematically investigated.

Therefore, in the present study, we integrated pan-cancer transcriptomic datasets, bulk RNA sequencing, single-cell RNA sequencing datasets, and clinical glioma specimens to comprehensively investigate the biological and clinical significance of *CAMK2A*. Specifically, we systematically characterized its expression pattern, prognostic value, cellular localization, and association with neuron-like differentiation programs, and further validated these findings using clinical glioma tissue samples.

## 2. Methods

### 2.1. Comparative Analysis of CAMK2A RNA Expression Across Multiple Tumor Types

*CAMK2A* RNA expression across The Cancer Genome Atlas (TCGA) cancer cohorts was analyzed using TIMER2.0 (https://compbio.cn/timer2/, accessed on 12 March 2026) [20,21,22]. RNA-sequencing data from TCGA tumor samples and available normal tissues were assessed through the Gene_DE module under the Exploration panel of the TIMER2.0 web server. *CAMK2A* was entered as the gene of interest, and differential expression plots were generated to compare *CAMK2A* transcript levels between tumor and normal tissues across cancer types.

Because normal tissue controls are unavailable for several cancer types in TCGA, we further performed an integrated analysis using the Sangerbox platform (http://sangerbox.com/home.html, accessed on 12 March 2026) [23]. According to the platform annotation, both TCGA RNA-sequencing data and Genotype-Tissue Expression (GTEx) normal tissue expression data used in this analysis were obtained from the UCSC Xena platform (https://xena.ucsc.edu/) [24,25]. *CAMK2A* RNA expression was analyzed using the uniformly processed expression matrix provided through Sangerbox based on UCSC Xena data. Expression levels were compared among TCGA tumor tissues, available TCGA normal tissues, and corresponding GTEx normal tissue controls across cancer types.

To more directly visualize differential *CAMK2A* expression between selected tumor types and normal tissues, GEPIA2 (http://gepia2.cancer-pku.cn/, accessed on 11 April 2026) was used to generate scatter plots of *CAMK2A* expression [26]. Transcript levels were displayed as transcripts per million (TPM) values without log transformation, allowing direct comparison of *CAMK2A* RNA expression between tumor and normal samples. GEPIA2 was further used to compare *CAMK2A* RNA expression between selected tumor types and corresponding normal tissues, and to evaluate *CAMK2A* expression across different pathological stages [26]. *CAMK2A* expression in glioma was further assessed using the Chinese Glioma Genome Atlas (CGGA) platform (https://www.cgga.org.cn/index.jsp, accessed on 29 April 2026) [27,28,29,30]. *CAMK2A* RNA expression levels were compared across glioma pathological subtypes and World Health Organization (WHO) grades using CGGA transcriptomic and clinicopathological data [27,28,29,30].

### 2.2. Comparative Analysis of CAMK2A Protein Expression Using CPTAC Proteomic Data

To evaluate whether altered *CAMK2A* RNA expression was reflected at the protein level, *CAMK2A* protein abundance was analyzed using CPTAC proteomic data through the UALCAN web platform (https://ualcan.path.uab.edu/, accessed on 12 March 2026) [31,32,33,34]. Tumor types with available CPTAC protein expression data and corresponding normal tissue controls were selected, and *CAMK2A* was entered as the protein of interest. Protein expression levels were compared between tumor and normal tissues using the platform-provided CPTAC datasets, and the resulting plots were used to assess differential *CAMK2A* protein expression between cancer and normal tissue samples.

### 2.3. Somatic Mutation Analysis of CAMK2A

Somatic alterations of *CAMK2A* across TCGA cancer cohorts were evaluated using TIMER3 (https://compbio.top/timer3/https://compbio.cn/timer3/, accessed on 12 March 2026) and Sangerbox (http://sangerbox.com/home.html, accessed on 4 May 2026) [21,22,23,35]. TIMER3 was used to summarize the mutation categories and alteration frequencies of *CAMK2A* across cancer types [21,22,35]. Sangerbox was further used to map *CAMK2A* mutation events to their corresponding genomic or protein positions and to visualize the site-specific distribution of mutations across tumor types [23]. These analyses were performed to characterize the mutation spectrum of *CAMK2A* in TCGA cancer cohorts.

To explore whether *CAMK2A* expression was associated with distinct genomic alteration patterns in glioma, mutation landscape analysis was performed using the Sangerbox platform (http://sangerbox.com/home.html, accessed on 4 May 2026) [23]. Glioma samples were stratified into *CAMK2A*-high and *CAMK2A*-low groups using the median *CAMK2A* RNA expression level as the cutoff. Somatic mutation profiles were compared between the two groups, and the most frequently mutated genes in each group were visualized using platform-generated mutation landscape plots. This analysis was used to characterize mutation patterns associated with differential *CAMK2A* expressions in glioma.

### 2.4. Survival Analysis of CAMK2 Family Genes and CAMK2A-Associated Prognostic Significance

The prognostic relevance of *CAMK2* family genes was first explored using GEPIA2 (http://gepia2.cancer-pku.cn/, accessed on 12 March 2026) [26]. *CAMK2* family members were entered as genes of interest, and survival-associated heatmaps were generated across cancer types using the survival analysis module provided by the platform. Tumor types showing significant associations between *CAMK2* family gene expression and patient survival were further selected for Kaplan–Meier survival analysis using the platform-generated survival plots.

To evaluate the prognostic relevance of *CAMK2A* methylation, survival analysis based on *CAMK2A* DNA methylation levels was performed using the Cancer Immunology Data Engine (CIDE; https://cide.ccr.cancer.gov/, accessed on 13 March 2026) [36]. For each available cancer type, patients were stratified according to *CAMK2A* methylation levels using the grouping strategy provided by the platform, and survival differences between methylation-defined groups were assessed using platform-generated survival analyses.

For glioma-specific validation, the association between *CAMK2A* RNA expression and overall survival was further examined in multiple independent glioma cohorts, including TCGA, REMBRANDT, CGGA693, CGGA325, and CGGA301 [27,28,29,30,37,38,39]. Expression and clinical survival data were integrated for each cohort, and samples lacking *CAMK2A* expression data or survival information were excluded. Patients were stratified into *CAMK2A*-high and *CAMK2A*-low groups according to *CAMK2A* RNA expression levels, using the optimal cutoff. To assess the robustness of the survival findings, a sensitivity analysis using the median *CAMK2A* expression as the cutoff was additionally performed and is presented in Supplementary Figure S1. Kaplan–Meier survival curves were generated in R software (version 4.1.2) using the survival and survminer packages, and survival differences between groups were assessed using the log-rank test. Hazard ratios and 95% confidence intervals were estimated using Cox proportional hazards models where applicable.

### 2.5. Protein Interaction, Functional Enrichment, and Co-Expression Analysis

A *CAMK2A*-centered protein-protein association network was constructed using the STRING database (https://string-db.org/, accessed on 13 March 2026) [40]. *CAMK2A* was entered as the query protein, and interacting proteins supported by experimental evidence, curated databases, co-expression, text mining, or other STRING evidence channels were retrieved using the platform settings. The resulting interaction network was used to identify *CAMK2A*-associated proteins. Functional annotation of these interacting proteins was further performed using the enrichment analysis function in STRING, including Gene Ontology (GO) biological process, cellular component, and molecular function categories, as well as Kyoto Encyclopedia of Genes and Genomes (KEGG) pathway analysis [41,42,43].

To identify genes transcriptionally associated with *CAMK2A* across cancer types, GEPIA2 (http://gepia2.cancer-pku.cn/, accessed on 13 March 2026) was used to screen genes showing the strongest expression correlations with *CAMK2A* in tumor datasets [26]. The top five *CAMK2A*-correlated genes were then selected for further visualization. TIMER2.0 (https://compbio.cn/timer2/, accessed on 13 March 2026) was used to generate correlation heatmaps showing the relationship between *CAMK2A* and these candidate genes across cancer types [20,21,22]. These analyses were performed to characterize *CAMK2A*-associated interaction networks, enriched biological functions, and co-expression patterns in tumor datasets.

### 2.6. Correlation Analysis of CAMK2A with Tumor-Related Molecular Features and RNA Modification Regulators

Correlation analyses between *CAMK2A* expression and tumor-related molecular features were performed using the Sangerbox platform (http://sangerbox.com/home.html, accessed on 29 April 2026) [23]. Across TCGA cancer cohorts, *CAMK2A* RNA expression was correlated with tumor purity, tumor mutational burden (TMB), microsatellite instability (MSI), homologous recombination deficiency (HRD), mutant-allele tumor heterogeneity (MATH), neoantigen load (NEO), loss of heterozygosity (LOH), and ploidy. Correlation coefficients and statistical significance were calculated using the platform-provided analysis module, and the results were visualized as cross-cancer correlation heatmaps.

Associations between *CAMK2A* expression and RNA modification regulators were further evaluated using Sangerbox (http://sangerbox.com/home.html, 29 April 2026) [23]. Genes involved in m1A, m5C, and m6A RNA modification pathways were included, and their expression correlations with *CAMK2A* were assessed across TCGA cancer cohorts. The resulting correlation heatmaps were used to characterize the relationship between *CAMK2A* and RNA modification-related regulatory programs across cancer types.

### 2.7. GSVA-Based Pathway Activity Analysis

Pathway activity was evaluated using gene set variation analysis (GSVA) in R [44]. The CGGA693 cohort was used for this analysis because it provided RNA-sequencing data together with relatively comprehensive clinicopathological and survival annotations [27,28,29,30]. Hallmark gene sets were imported using the GSEABase package, and sample-level GSVA scores were calculated using the GSVA package [45,46]. Glioma samples were stratified into *CAMK2A*-high and *CAMK2A*-low groups according to *CAMK2A* expression. GSVA score distributions across Hallmark pathways were visualized as heatmaps using ComplexHeatmap and circlize, with samples ordered by *CAMK2A* expression group and *CAMK2A* expression level [47,48,49]. Differences in pathway activity between *CAMK2A*-high and *CAMK2A*-low groups were further assessed using the limma package, and adjusted *p* values were calculated to account for multiple testing [50]. Differential pathway activity was visualized using volcano plots generated with ggplot2 and ggrepel [51,52].

### 2.8. ssGSEA-Based Cell-Type-like Signature Scoring

Cell-type-like enrichment scores were calculated using single-sample gene set enrichment analysis (ssGSEA) implemented in the GSVA package [44,53]. This analysis was performed using the CGGA693 cohort to maintain consistency with the pathway and regression analyses [28,29,30]. Marker gene sets were manually defined to represent neuron-like, astrocyte-like, mature oligodendrocyte-like, oligodendrocyte precursor cell-like, and pericyte-like signatures. The neuron-like signature included RBFOX3, SNAP25, and SYT1; the astrocyte-like signature included ALDH1L1, AQP4, GFAP, SLC1A2, and SLC1A3; the mature oligodendrocyte-like signature included MBP, MOG, and PLP1; the OPC-like signature included PDGFRA, SOX10, OLIG2, and CSPG4; and the pericyte-like signature included PDGFRB, RGS5, and NOTCH3 [54,55,56,57,58]. Associations between *CAMK2A* expression and these cell-type-like enrichment scores were assessed using Spearman correlation analysis with Benjamini–Hochberg false discovery rate correction [59]. Scatter plots and related visualizations were generated using ggplot2 [51]. Marker genes were selected based on previously reported brain cell-type and tumor microenvironment marker resources, and the resulting scores were interpreted as cell-type-like or proxy transcriptional enrichment scores rather than direct estimates of cell proportions.

### 2.9. Non-Tumor Microenvironment-Related Transcriptional Score Analysis

To account for broad non-tumor transcriptional signals in bulk glioma samples, stromal and immune proxy scores were calculated using ssGSEA implemented in the GSVA package [44,53]. The same CGGA693 cohort was used so that the resulting non-tumor proxy score could be incorporated into subsequent multivariable regression models [28,29,30]. The stromal proxy signature included *COL1A1, COL1A2, COL3A1, DCN, LUM, TAGLN, THY1*, and *VIM* [60,61,62]. The immune proxy signature included *PTPRC, CD3D, CD3E, CD14, TYROBP, LST1, HLA-DRA,* and *AIF1* [63]. These marker genes were selected based on previously reported tumor microenvironment and brain cell marker resources [60,61,62,63]. The stromal and immune scores were interpreted as proxy transcriptional enrichment scores rather than direct estimates of stromal or immune cell proportions [64,65]. The two scores were standardized and summed to generate a non-tumor proxy score, which was used as an adjustment variable in subsequent multivariable regression analyses to reduce potential confounding from stromal and immune transcriptional components [64,65].

To evaluate the association between *CAMK2A* expression and tumor microenvironment-related transcriptional signals in glioma, correlation analyses were performed using the Sangerbox platform (http://sangerbox.com/home.html, accessed on 29 April 2026) [23]. UCSC Xena-normalized TCGA RNA-sequencing datasets were used, and samples from the TCGA GBM, GBMLGG, and LGG cohorts were extracted for analysis [24,37,38]. For each cohort, ESTIMATE scores, immune scores, and stromal scores were calculated using the ESTIMATE R package as implemented by the platform [66]. Correlations between *CAMK2A* expression and each ESTIMATE-derived score were then assessed and visualized as scatter plots.

### 2.10. Multivariable Cox and Linear Regression Analyses

Multivariable regression analyses were performed in R to evaluate the independent associations among *CAMK2A* expression, clinicopathological variables, patient survival, pathway activity, and cell-type-like enrichment scores. These models were constructed using the CGGA693 cohort by integrating matched *CAMK2A* expression data, clinicopathological and survival annotations, and the derived GSVA, ssGSEA, and non-tumor proxy scores where applicable [28,29,30]. Clinical and expression data were processed using data.table, dplyr, stringr, and edgeR [67,68,69]. For survival outcomes, univariable and multivariable Cox proportional hazards regression models were constructed using the survival package to assess the association of *CAMK2A* expression and clinicopathological variables with overall survival [70,71]. Hazard ratios, 95% confidence intervals, and *p* values were extracted using the broom package, and Cox regression results were visualized as forest plots using ggplot2 [51].

For pathway-level analysis, multivariable linear regression models were constructed using base R functions to assess the association between *CAMK2A* expression and HALLMARK_KRAS_SIGNALING_DN GSVA scores after adjustment for available clinicopathological variables [44,45]. Regression coefficients, 95% confidence intervals, and *p* values were extracted from model outputs and visualized as forest plots using ggplot2 [51].

For cell-type-like signature analysis, multivariable linear regression models were used to evaluate the association between *CAMK2A* expression and ssGSEA-derived cell-type-like enrichment scores after adjustment for available clinical variables and non-tumor proxy scores [44,53]. Regression coefficients and 95% confidence intervals for *CAMK2A* were extracted using broom and visualized as forest plots using ggplot2 [51,72]. Samples with missing values for variables included in each model were excluded from the corresponding analysis.

### 2.11. Single-Cell Transcriptomic Visualization Using Public Glioma Datasets

*CAMK2A* expression at the single-cell level was examined using a publicly available single-nucleus RNA-sequencing dataset from the Broad Institute Single Cell Portal (https://singlecell.broadinstitute.org/single_cell, accessed on 11 April 2026) [73]. The dataset, “Tumor Transcriptional Subtype Is a Key Axis of Immune Checkpoint Blockade Response in Glioblastoma” (accession: SCP3341), was derived from immune-checkpoint-blockade-treated IDH-wildtype glioblastoma samples reported by Ghannam et al. [74]. Platform-provided visualization tools were used to generate UMAP plots showing *CAMK2A* expression and study-annotated cellular populations. *CAMK2A* expression across annotated cell populations was further visualized using platform-generated violin plots. The processed expression data, UMAP coordinates, and cell-type annotations provided by the original study were used directly, without raw data reprocessing, reclustering, or de novo cell-type annotation. The primary single-cell results were based on the UMI-based snRNA-seq dataset SCP3341, using the processed expression data, UMAP coordinates, and cell annotations provided by the original study.

As a complementary analysis, single-cell expression visualization was further performed using the publicly available IDH-mutant glioma dataset GSE227718 from the Gene Expression Omnibus (GEO; https://www.ncbi.nlm.nih.gov/geo/, accessed on 16 April 2026) [75,76]. A processed TPM expression matrix from this dataset was imported into R using the data.table package and formatted for downstream single-cell analysis [68]. The matrix was converted into a sparse matrix using the Matrix package and used to construct a Seurat object with the CreateSeuratObject function in the Seurat package [77,78]. Quality-control metrics, including the number of detected genes per cell and the percentage of mitochondrial transcripts, were calculated in Seurat. Cells were retained using conservative filtering thresholds based on detected gene number and mitochondrial transcript percentage. Because the input matrix consisted of processed TPM values rather than raw UMI counts, this analysis was used for exploratory expression visualization rather than quantitative cell abundance estimation. This dataset was therefore used only as a complementary analysis and did not form the basis of the primary single-cell findings. After quality control, highly variable features were identified using the FindVariableFeatures function, followed by data scaling, principal component analysis, nearest-neighbor graph construction, clustering, and UMAP dimensionality reduction using Seurat. UMAP feature plots were generated with the FeaturePlot function to visualize the expression patterns of *CAMK2A* and selected representative marker genes, including MAP2 as a neuronal marker, GFAP as a glial/glioma-associated marker, and TYROBP as an immune/myeloid marker [54,63,79]. Plots were further formatted using ggplot2 where applicable [51].

### 2.12. Protein Expression Visualization Using the Human Protein Atlas

*CAMK2A* protein expression across cancer types was further examined using the Human Protein Atlas (HPA) Cancer resource (https://www.proteinatlas.org/, accessed on 4 May 2026) [80,81,82,83,84,85,86,87,88,89]. The protein expression summary plot was obtained from the HPA *CAMK2A* cancer page using antibody HPA051783. In this summary, color-coded bars indicate the percentage of patients with high or medium *CAMK2A* protein expression for each cancer type, whereas low or undetected protein expression is shown as a white bar. This analysis was based on HPA antibody-based immunohistochemistry annotations and was used as a descriptive visualization of *CAMK2A* protein expression across cancer tissues.

Representative immunohistochemistry images of *CAMK2A* staining were also obtained from the HPA Cancer resource. Four tumor tissue images were selected from the platform-provided image and annotation pages, including two images from patient ID 122 stained with antibody HPA051783 and two images from patient ID 1599 stained with antibody CAB004330. These images were used for illustrative visualization of *CAMK2A* protein staining patterns and were not re-scored or quantitatively reanalyzed. HPA-derived images and data were used and cited according to the HPA license and citation guidelines.

### 2.13. Immunotherapy Response Analysis Using Public Glioma Treatment Cohorts

To explore the potential association between *CAMK2A* expression and immunotherapy response in glioma, public treatment cohorts were analyzed using the Tumor Immunotherapy Gene Expression Resource (TIGER; http://tiger.canceromics.org/, accessed on 4 May 2026) [90]. *CAMK2A* was queried in the Immunotherapy Response module to compare its expression across available immunotherapy datasets. A cross-cohort differential expression volcano plot was generated by the platform to summarize *CAMK2A* expression differences between responder and non-responder groups across datasets [90,91,92].

Based on this screening, the GBM-PRJNA482620 anti-PD-1 cohort was selected for detailed analysis [90,92]. *CAMK2A* was entered in the Immunotherapy Response module for this cohort, and platform-generated plots were obtained to compare *CAMK2A* expression between responders and non-responders. The association between *CAMK2A* expression and overall survival in this cohort was further evaluated using TIGER platform-derived Cox regression analysis and visualized as a forest plot. These analyses were based on the processed expression and clinical response annotations provided by TIGER, without additional reprocessing of the original transcriptomic data.

### 2.14. Multiplex Immunohistochemistry Staining and Image Acquisition

Tumor tissues and gliosis tissues were obtained from Xiangya Hospital of Central South University. Tissue collection and experimental procedures were approved by the Medical Ethics Committee of Xiangya Hospital of Central South University (Approval number: 202401003). All experimental procedures were carried out in compliance with the official relevant guidelines and regulations.

Multiplex immunofluorescence (mIF) staining was performed on 4-μm formalin-fixed paraffin-embedded (FFPE) sections from gliosis tissues and glioma specimens using a tyramide signal amplification (TSA)-based cyclic staining system (Aifang Bio, Changsha, China; AFIHC025). Briefly, tissue sections were deparaffinized, rehydrated through graded ethanol, and sequentially incubated with primary antibodies against GFAP (1:500, AFRM0041, Aifang Bio, Changsha, China), MAP2 (1:500, AFRM0213, Aifang Bio, Changsha, China), and CaMKII (1:300, AFRM0099, Aifang Bio, Changsha, China). Following incubation with HRP-conjugated secondary antibodies, TSA fluorophore deposition was performed using TYR-520 (Aifang Bio, Changsha, China) for GFAP, TYR-620 for MAP2, and TYR-570 for CaMKII. After each staining cycle, heat-induced antibody stripping was carried out to remove bound primary and secondary antibodies while preserving the covalently deposited tyramide fluorescence signals for subsequent rounds of staining. After completion of all staining cycles, nuclei were counterstained with DAPI, and the sections were mounted with antifade mounting medium. Multispectral fluorescence images were acquired using the VECTRA Polaris Automated Quantitative Pathology Imaging System (Akoya Biosciences, Marlborough, MA, USA). Fluorescence image analysis and quantitative assessment of fluorescence intensity were performed using QuPath (version 0.7.0) and ImageJ (version 1.53c).

## 3. Results

### 3.1. Pan-Cancer Multi-Omics Characterization of CAMK2A

**Figure 1:**
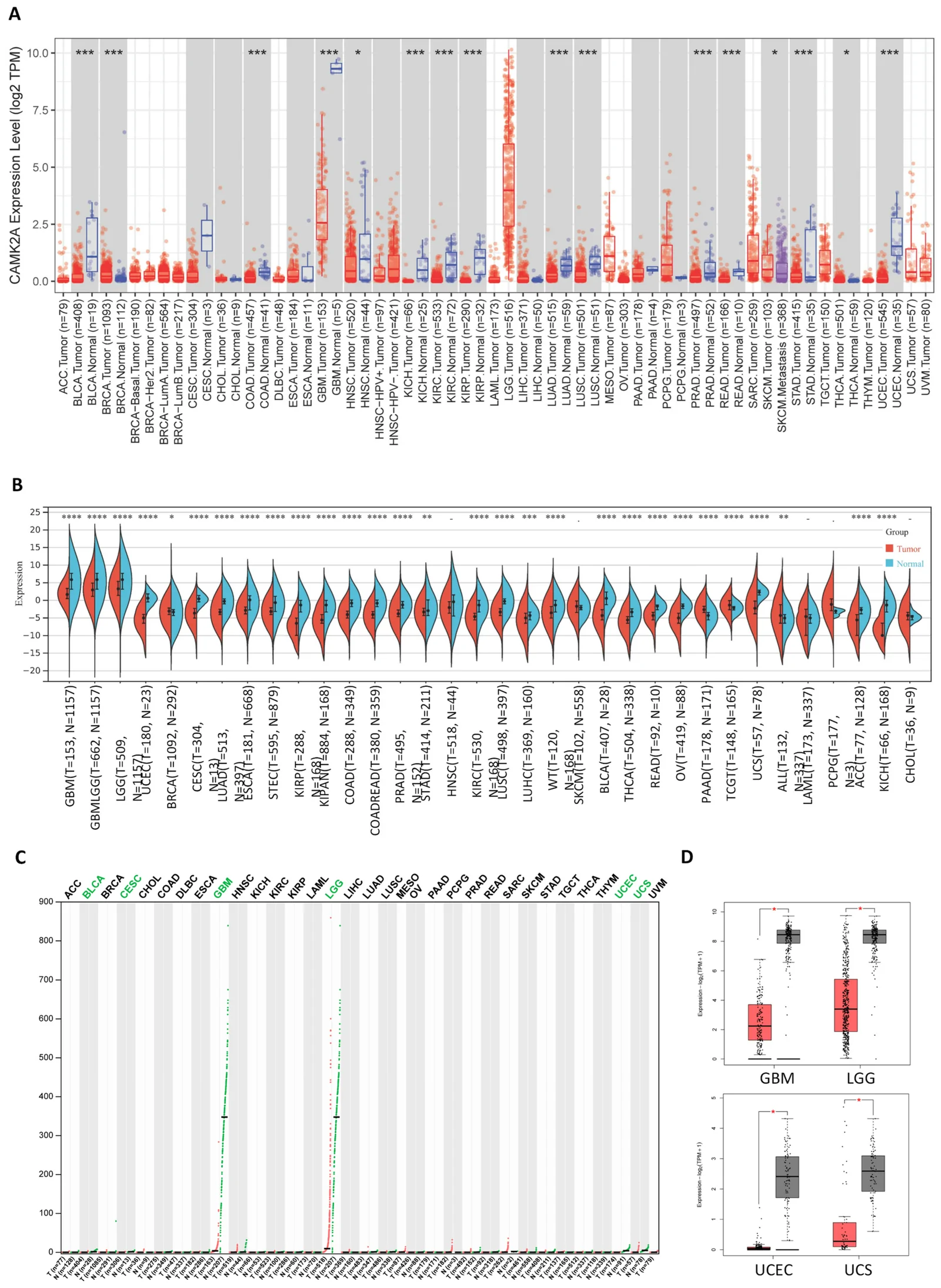
Pan-cancer expression landscape of *CAMK2A*. (**A**) Differential *CAMK2A* mRNA expression between tumor and normal tissues across TCGA datasets. (**B**) Comparison of *CAMK2A* expression across multiple cancer types using GTEx datasets. (**C**) *CAMK2A* expression distribution in pan-cancer datasets displayed on a linear expression scale. (**D**) Validation of *CAMK2A* downregulation in glioblastoma (GBM), lower-grade glioma (LGG), uterine corpus endometrial carcinoma (UCEC), and uterine carcinosarcoma (UCS). * *p* < 0.05; ** *p* < 0.01; *** *p* < 0.001; **** *p* < 0.0001.

To systematically characterize the molecular landscape of *CAMK2A* across human cancers, we first analyzed its transcriptomic expression patterns using The Cancer Genome Atlas (TCGA) datasets. Comparisons between tumor tissues and matched normal controls available within TCGA revealed widespread alterations in *CAMK2A* expression across multiple cancer types (Figure 1A). However, several tumor entities lack corresponding normal tissues in TCGA, limiting comprehensive tumor–normal comparisons. To address this limitation, transcriptomic data from the Genotype-Tissue Expression (GTEx) project were incorporated to provide additional normal tissue references and enable a more complete pan-cancer analysis (Figure 1B). Integration of GTEx normal tissue datasets substantially expanded the range of tumor types available for comparison and confirmed broad dysregulation of *CAMK2A* across human malignancies (Figure 1B). Although the magnitude and direction of expression changes varied among cancer types, multiple tumors exhibited marked reductions in *CAMK2A* expressions relative to their corresponding normal tissues. To facilitate visualization of these differences, *CAMK2A* expression levels were subsequently displayed on a linear scale without logarithmic transformation. This analysis highlighted substantial inter-tumor heterogeneity and identified glioblastoma (GBM) and lower-grade glioma (LGG) among the tumor types exhibiting the most pronounced reductions in *CAMK2A* expression (Figure 1C). Direct quantitative comparisons further confirmed significant downregulation of *CAMK2A* in both GBM and LGG. Similar decreases were also observed in uterine corpus endometrial carcinoma (UCEC) and uterine carcinosarcoma (UCS) (Figure 1D).

**Figure 2:**
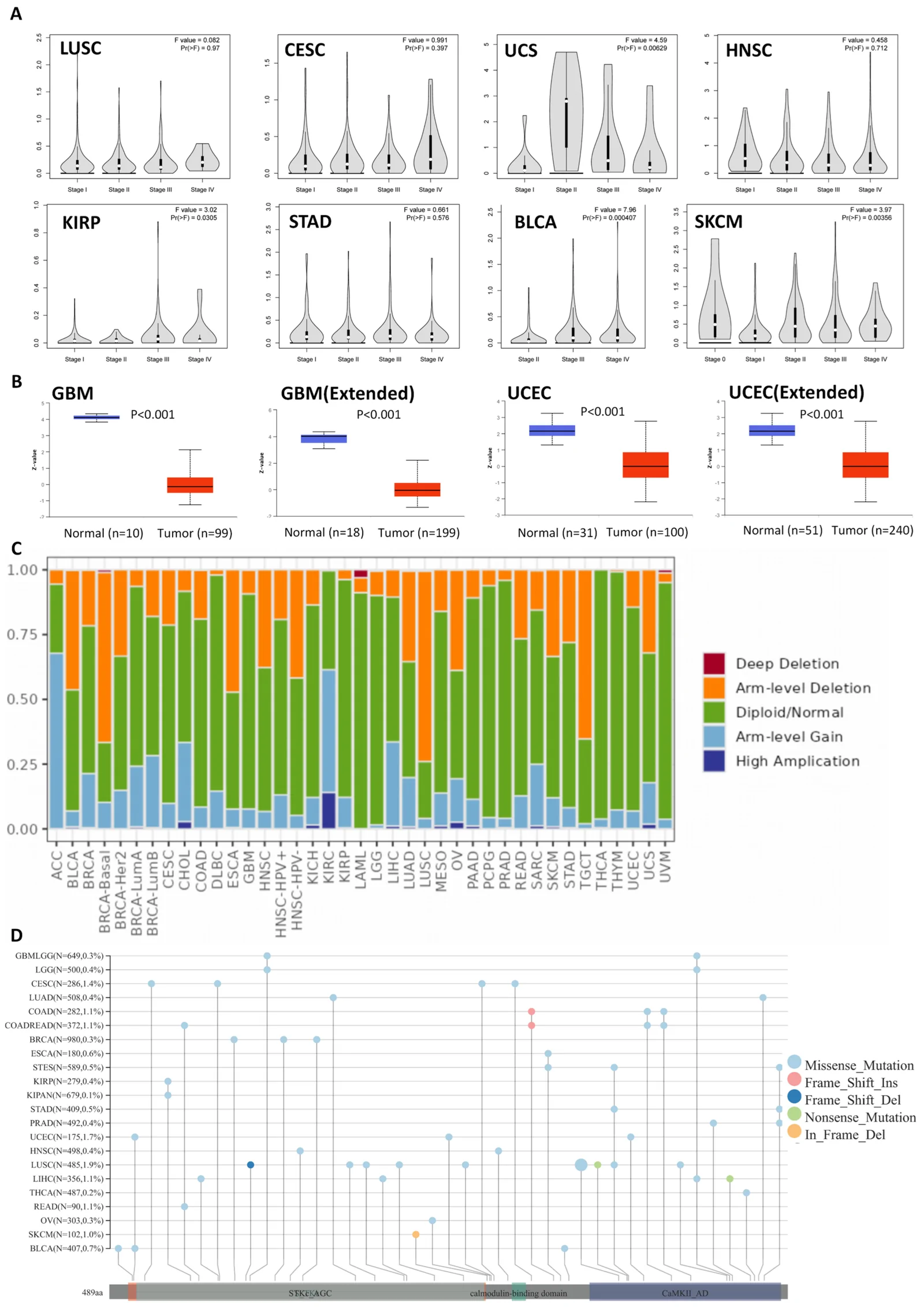
Genomic and proteomic characteristics of *CAMK2A* across human cancers. (**A**) Association between *CAMK2A* expression and pathological stage in selected cancer types. (**B**) Validation of *CAMK2A* protein expression in GBM and UCEC using CPTAC standard and expanded cohorts. (**C**) Copy number variation (CNV) profiles of *CAMK2A* across TCGA cancer types. (**D**) Mutation landscape of *CAMK2A* across human cancers, showing mutation frequencies and mutation types.

To investigate the potential clinical relevance of *CAMK2A* across cancers, we next evaluated its association with pathological stage. Significant stage-dependent differences in *CAMK2A* expression were observed in uterine carcinosarcoma (UCS), kidney renal papillary cell carcinoma (KIRP), bladder urothelial carcinoma (BLCA), and skin cutaneous melanoma (SKCM), whereas no significant associations were detected in several other tumor types (Figure 2A). These findings suggest a potential relationship between *CAMK2A* expression and tumor progression in selected malignancies. To determine whether the observed transcriptomic alterations were reflected at the protein level, we analyzed proteomic datasets from the Clinical Proteomic Tumor Analysis Consortium (CPTAC). Consistent with the mRNA expression profiles, CAMKII protein abundance was significantly reduced in GBM tissues compared with normal brain tissues (Figure 2B). A similar decrease was also observed in UCEC tumor samples relative to corresponding normal controls (Figure 2B), indicating concordant downregulation of *CAMK2A* at both transcriptomic and proteomic levels.

We next explored the genomic alteration landscape of *CAMK2A* across TCGA cancer cohorts. Copy number analysis revealed that diploid/normal status represented the predominant genomic state in most tumor types (Figure 2C). Among the detected alterations, arm-level gains and arm-level deletions were the most frequent events, whereas deep deletions and high-level amplifications occurred only rarely. Consistent with these findings, somatic mutations of *CAMK2A* were uncommon across cancers, with mutation frequencies generally below 2% (Figure 2D). Missense mutations represented the predominant mutation category, whereas frameshift insertions, frameshift deletions, nonsense mutations, and in-frame deletions were detected only sporadically. Furthermore, mutations were distributed throughout the coding sequence, including the serine/threonine kinase catalytic domain and the CaMKII association domain, without evidence of recurrent hotspot regions (Figure 2D).

Collectively, these findings demonstrate that *CAMK2A* is broadly dysregulated across human malignancies at both the transcriptomic and proteomic levels while exhibiting relatively infrequent genomic alterations.

### 3.2. CAMK2A Exhibits Prognostic Significance and Is Associated with Neuronal Signaling Networks

**Figure 3:**
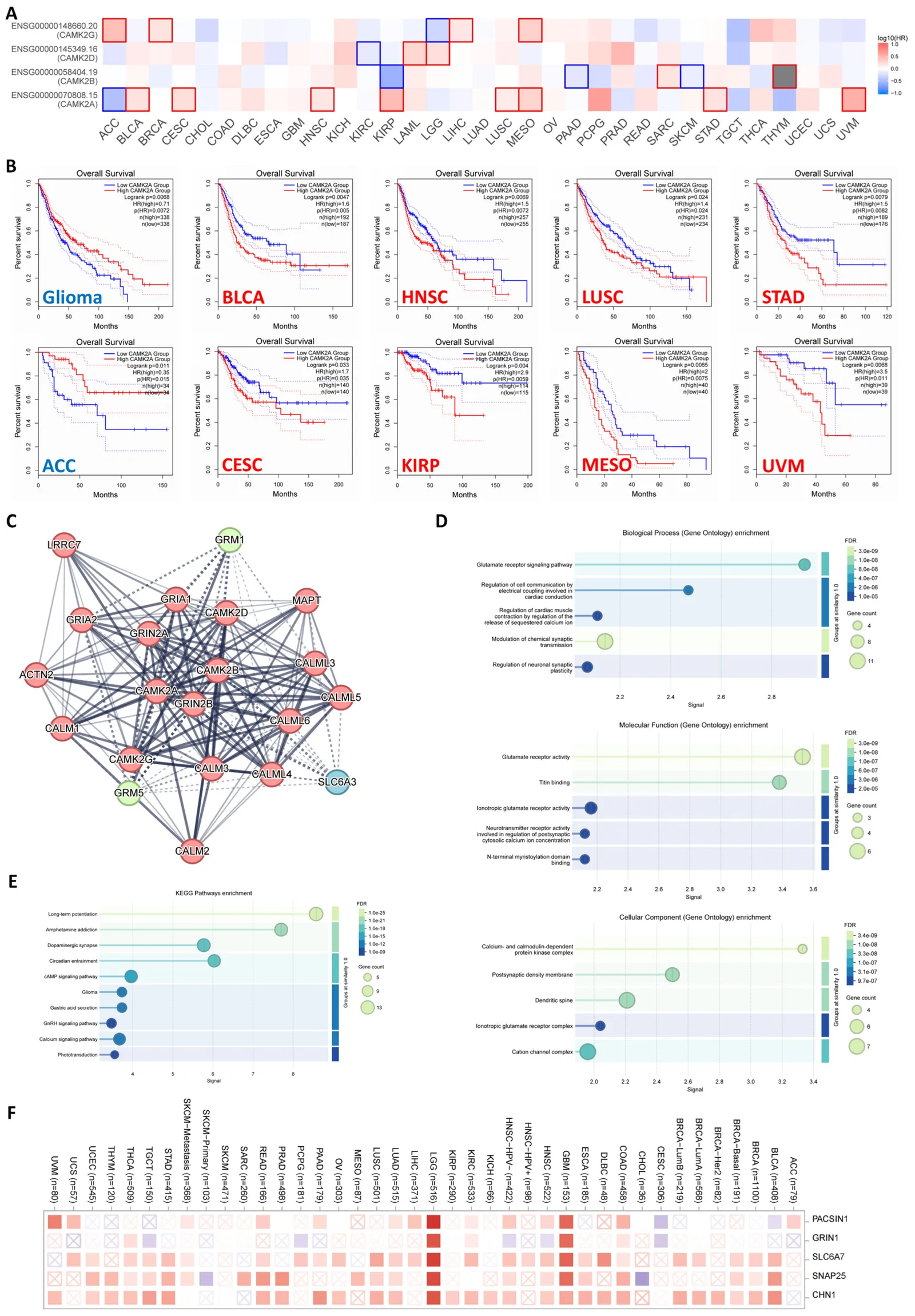
Prognostic significance and functional characteristics of *CAMK2A* across cancers. (**A**) Univariate Cox regression analysis evaluating the prognostic significance of CAMK2 family members across TCGA cancer types. (**B**) Kaplan-Meier survival analyses of overall survival according to *CAMK2A* expression in representative cancers. (**C**) Protein-protein interaction (PPI) network of *CAMK2A*-associated proteins. (**D**) Gene Ontology (GO) enrichment analysis of *CAMK2A*-related genes. (**E**) KEGG pathway enrichment analysis of *CAMK2A*-related genes. (**F**) Correlation analysis between *CAMK2A* and representative neuron-associated genes across multiple cancer types.

To evaluate the prognostic significance of *CAMK2* family members, a pan-cancer survival analysis was performed for *CAMK2A*, *CAMK2B*, *CAMK2D*, and *CAMK2G*. Significant associations between gene expression and overall survival were observed across multiple tumor types (Figure 3A). Among the four family members, *CAMK2A* exhibited the most prominent prognostic associations. Elevated *CAMK2A* expression was associated with shorter overall survival in bladder urothelial carcinoma (BLCA), head and neck squamous cell carcinoma (HNSC), lung squamous cell carcinoma (LUSC), stomach adenocarcinoma (STAD), cervical squamous cell carcinoma and endocervical adenocarcinoma (CESC), kidney renal papillary cell carcinoma (KIRP), mesothelioma (MESO), and uveal melanoma (UVM), whereas an inverse association was observed in adrenocortical carcinoma (ACC) (Figure 3A). Kaplan–Meier survival analyses further validated the prognostic relevance of *CAMK2A* in these tumor types (Figure 3B). High *CAMK2A* expression was associated with favorable overall survival in glioma and ACC, whereas poorer survival outcomes were observed in BLCA, HNSC, LUSC, STAD, CESC, KIRP, MESO, and UVM patients with elevated *CAMK2A* expression.

To explore the biological context of *CAMK2A*, a protein–protein interaction (PPI) network was constructed using experimentally validated and literature-supported interacting proteins (Figure 3C). The resulting network comprised multiple proteins involved in synaptic transmission, glutamatergic signaling, calcium homeostasis, and calmodulin-dependent signaling.

Gene Ontology enrichment analysis revealed that *CAMK2A*-associated proteins were predominantly enriched in neuronal and synaptic processes, including glutamate receptor signaling, modulation of chemical synaptic transmission, and regulation of neuronal synaptic plasticity (Figure 3D). Molecular function analysis highlighted glutamate receptor-related activities and neurotransmitter receptor functions involved in postsynaptic calcium regulation, while cellular component enrichment identified postsynaptic density membranes, dendritic spines, ionotropic glutamate receptor complexes, and calcium/calmodulin-dependent protein kinase complexes as the major enriched categories (Figure 3D).

Consistent with the GO results, KEGG pathway analysis demonstrated significant enrichment in neuronal signaling pathways, including long-term potentiation, dopaminergic synapse, circadian entrainment, cAMP signaling, and calcium signaling (Figure 3E). Additional enrichment was observed in glioma-related pathways, as well as GnRH signaling and phototransduction pathways.

Correlation analysis further identified *PACSIN1, GRIN1, SLC6A7, SNAP25,* and *CHN1* as the genes most strongly associated with *CAMK2A* expression across TCGA cancer cohorts (Figure 3F). Notably, all five genes exhibited strong positive correlations with *CAMK2A* in both GBM and LGG, highlighting a particularly tight transcriptional association within glioma. Similar positive correlations were also observed across multiple other cancer types, although the strength of these associations varied among tumors. Importantly, all five genes are functionally involved in neuronal and synaptic processes, including synaptic vesicle trafficking, neurotransmitter transport, and neuronal signaling, consistent with the GO and KEGG enrichment results.

### 3.3. CAMK2A Is Associated with Tumor Genomic Features, RNA Modification Regulators, and Methylation-Related Prognosis

**Figure 4:**
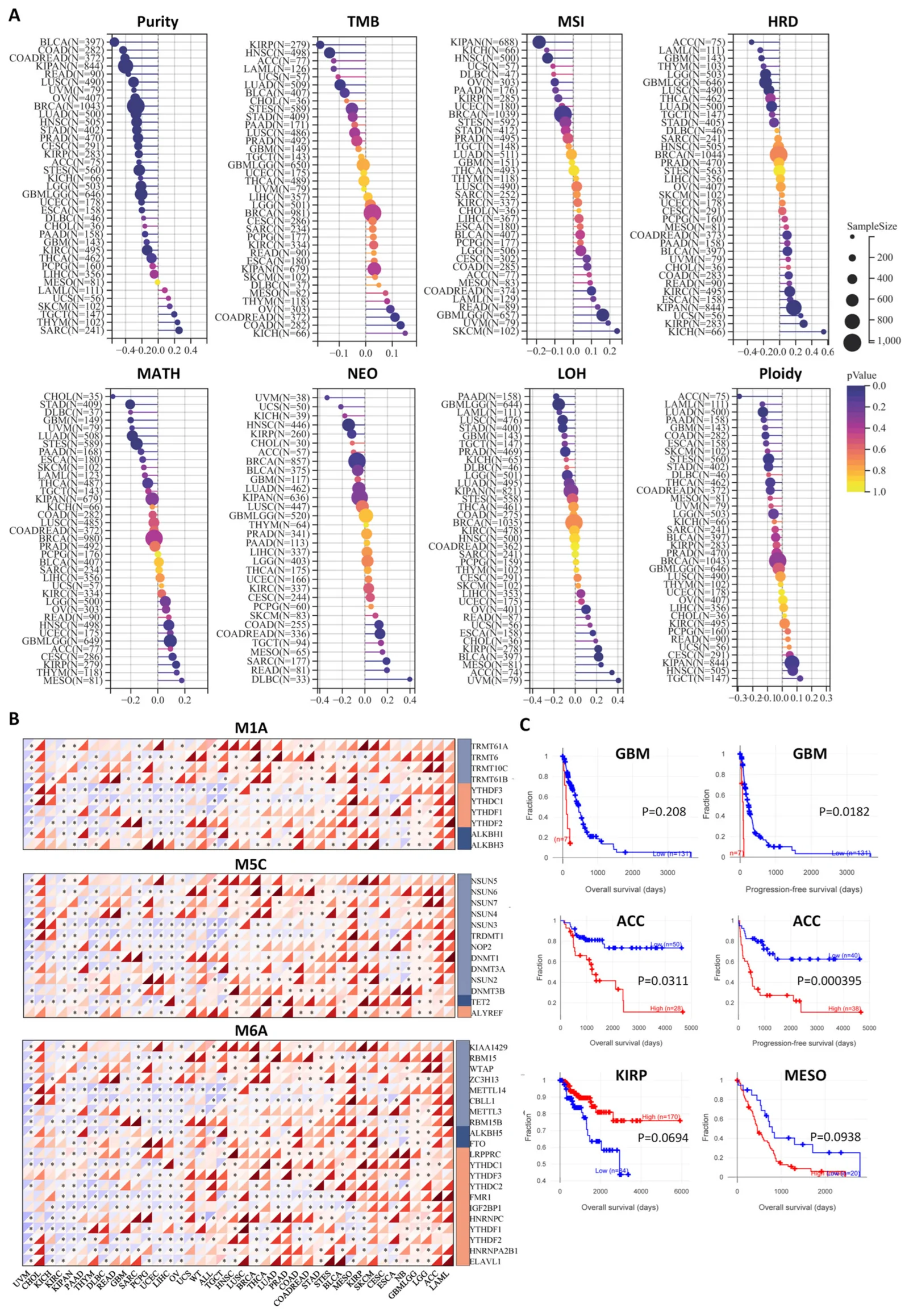
Molecular characteristics associated with CAMK2A across cancers. (**A**) Correlations between CAMK2A expression and tumor purity, tumor mutational burden (TMB), microsatellite instability (MSI), homologous recombination deficiency (HRD), mutant-allele tumor heterogeneity (MATH), neoantigen load (NEO), loss of heterozygosity (LOH), and tumor ploidy across cancers. (**B**) Correlation heatmaps between CAMK2A expression and RNA modification regulators, including m^1^A-, m^5^C-, and m^6^A-related genes. (**C**) Kaplan–Meier survival analyses according to CAMK2A DNA methylation status in representative cancer types. * *p* < 0.05.

To further characterize the molecular features associated with *CAMK2A*, correlations between *CAMK2A* expression and multiple tumor-related characteristics were evaluated across TCGA cancer cohorts, including tumor purity, tumor mutational burden (TMB), microsatellite instability (MSI), homologous recombination deficiency (HRD), mutant-allele tumor heterogeneity (MATH), neoantigen load (NEO), loss of heterozygosity (LOH), and ploidy (Figure 4A). Distinct correlation patterns were observed across cancer types, highlighting substantial heterogeneity in the relationships between *CAMK2A* expression and genomic or microenvironmental features.

We next examined the association between *CAMK2A* expression and RNA modification regulators. Correlation analyses revealed widespread relationships between *CAMK2A* and genes involved in m1A, m5C, and m6A RNA modification pathways across multiple tumor types (Figure 4B). Both positive and negative correlations were identified, although their magnitude and direction varied considerably among cancer cohorts.

To investigate whether epigenetic regulation of *CAMK2A* is associated with patient outcomes, we analyzed the prognostic significance of *CAMK2A* methylation across multiple cancer types (Figure 4C). In GBM, patients with high *CAMK2A* methylation exhibited markedly worse clinical outcomes than those with low methylation, as reflected by the substantially steeper decline of the survival curves. Despite the limited number of methylated GBM cases (*n* = 7), *CAMK2A* methylation was significantly associated with shorter progression-free survival (*p* = 0.0182), whereas a similar but non-significant trend was observed for overall survival. Importantly, these findings are consistent with our previous observation that reduced *CAMK2A* expression predicts unfavorable prognosis in glioma. However, given the very small number of methylated GBM cases (*n* = 7) and the resulting limited statistical power, this observation should be interpreted with caution and requires validation in larger cohorts. Significant methylation-associated survival differences were also observed in ACC, whereas no significant associations were detected in KIRP or MESO (Figure 4C).

### 3.4. CAMK2A Is an Independent Prognostic Marker Associated with Glioma Grade and Molecular Features

**Figure 5:**
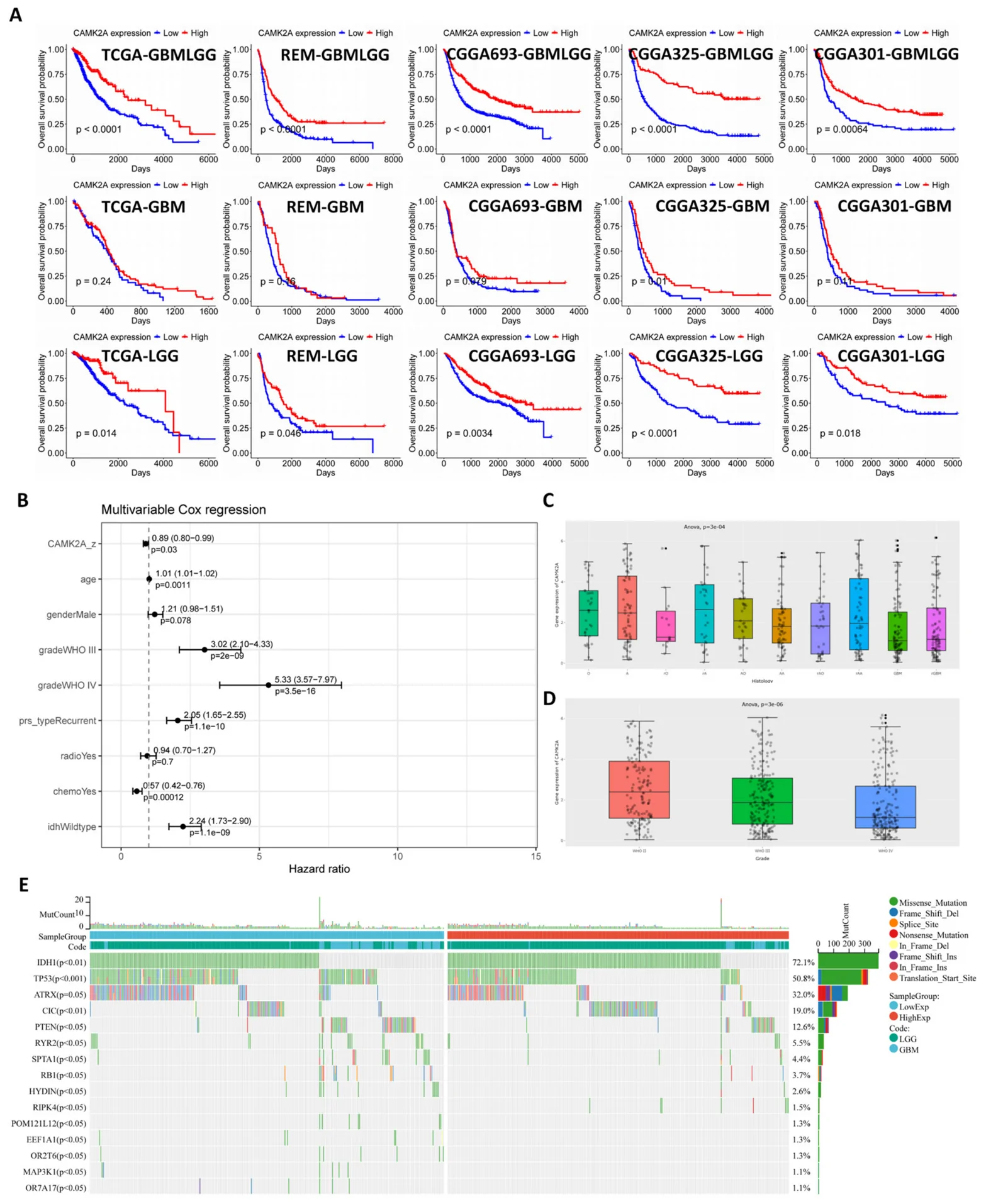
Clinical significance of *CAMK2A* in diffuse glioma. (**A**) Kaplan-Meier survival analyses of *CAMK2A* expression in TCGA, Rembrandt, CGGA693, CGGA325, and CGGA301 glioma cohorts. Analyses were performed in combined glioma (GBMLGG), GBM, and LGG cohorts. (**B**) Multivariable Cox regression analysis evaluating the independent prognostic value of *CAMK2A* after adjustment for clinicopathological and molecular variables. (**C**,**D**) Associations between *CAMK2A* expression and histological subtype and WHO grade. (**E**) Somatic mutation landscapes of gliomas with high and low *CAMK2A* expression.

Kaplan–Meier survival analyses were performed across multiple independent glioma cohorts to assess the prognostic significance of *CAMK2A* expression. Elevated *CAMK2A* expression was consistently associated with prolonged overall survival in the TCGA-GBMLGG cohort and four independent validation datasets, including Rembrandt, CGGA693, CGGA325, and CGGA301 (Figure 5A). Importantly, this association was validated across both Western and East Asian populations, highlighting the robustness and broad applicability of *CAMK2A* as a prognostic marker in glioma. Similar survival benefits were observed in individual LGG cohorts, further supporting the favorable prognostic value of *CAMK2A* in diffuse gliomas (Figure 5A). Sensitivity analyses using the median *CAMK2A* expression as the cutoff showed an overall consistent association between higher *CAMK2A* expression and better survival, although statistical significance was not reached in some subgroup analyses (Supplementary Figure S1).

To determine whether *CAMK2A* possesses independent prognostic value, multivariable Cox regression analysis was performed. After adjustment for age, sex, WHO grade, recurrence status, radiotherapy, chemotherapy, and IDH mutation status, *CAMK2A* expression remained significantly associated with overall survival, indicating that its prognostic effect was independent of established clinical and molecular factors (Figure 5B).

*CAMK2A* expression differed significantly among glioma histological subtypes (Figure 5C). Moreover, *CAMK2A* expression progressively declined with increasing WHO grade, reaching its lowest level in grade IV gliomas (Figure 5D).

To further explore the molecular characteristics associated with *CAMK2A* expression, mutational landscapes were compared between the *CAMK2A*-high and *CAMK2A*-low groups (Figure 5E). Gliomas with high *CAMK2A* expression were enriched for mutations in IDH1, TP53, ATRX, and CIC, whereas PTEN alterations occurred more frequently in the *CAMK2A*-low group. These findings suggest that *CAMK2A* expression is closely linked to distinct molecular subtypes and genomic features of glioma.

### 3.5. CAMK2A-High Gliomas Exhibit Attenuated Malignant Signaling and Neuronal Transcriptional Signatures

**Figure 6:**
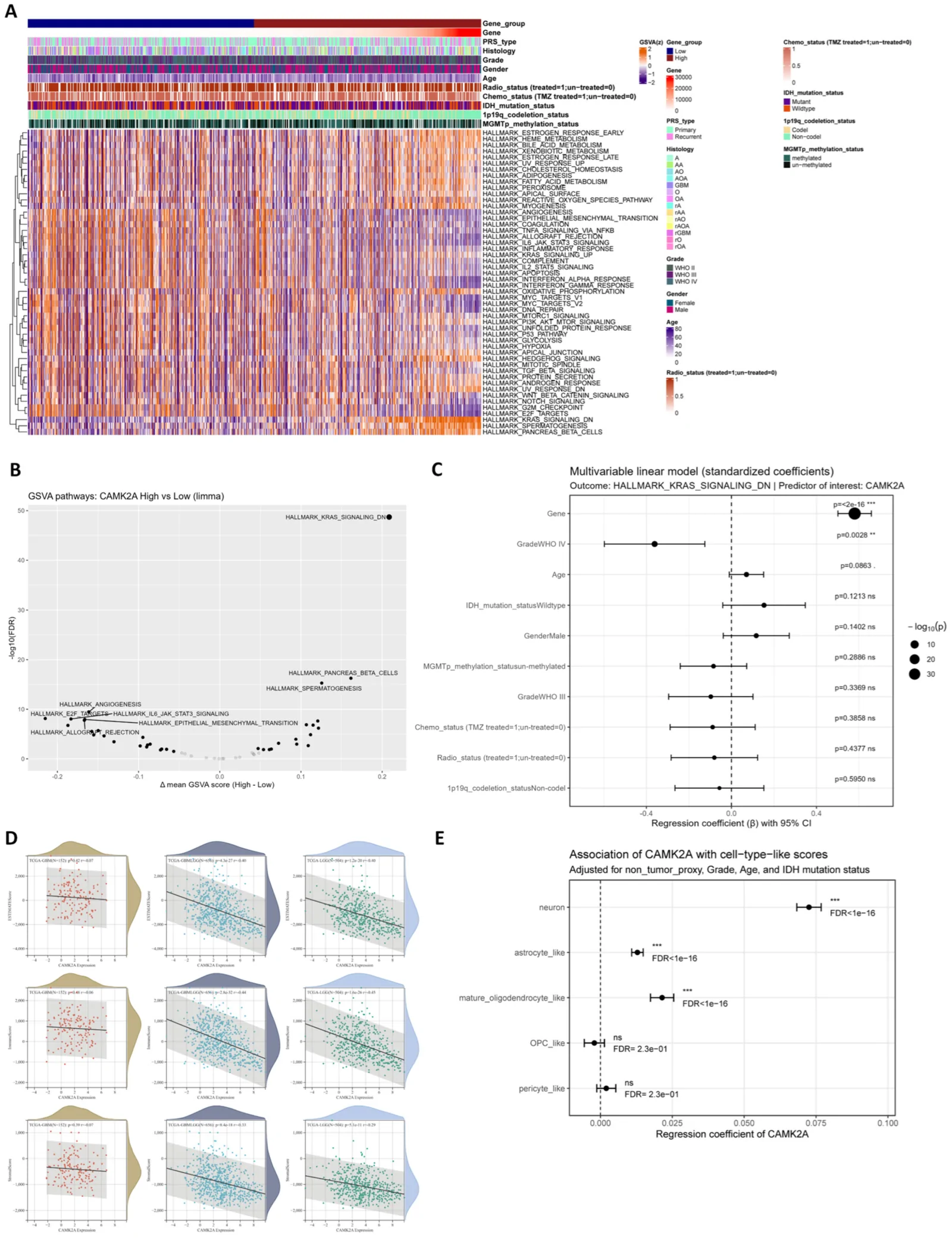
CAMK2A is associated with neuron-like transcriptional programs in glioma. (**A**) GSVA heatmap showing pathway activities in gliomas stratified by CAMK2A expression. (**B**) Differentially enriched Hallmark pathways between CAMK2A-high and CAMK2A-low gliomas. (**C**) Multivariable linear regression analysis evaluating the independent association between CAMK2A expression and the Hallmark KRAS signaling DN pathway. (**D**) Associations between CAMK2A expression and ESTIMATE-derived stromal, immune, and ESTIMATE scores. (**E**) Associations between CAMK2A expression and cell-type-like transcriptional signatures evaluated by ssGSEA. ** *p* < 0.01, *** *p* < 0.001; ns, not significant.

To characterize biological pathways associated with *CAMK2A* expression, GSVA was performed using Hallmark gene sets in glioma samples. Unsupervised clustering of GSVA scores revealed distinct pathway activity patterns between *CAMK2A*-high and *CAMK2A*-low tumors (Figure 6A). Differential pathway analysis identified HALLMARK_KRAS_SIGNALING_DN as the most significantly altered pathway between the two groups (Figure 6B). In contrast, several pathways implicated in tumor progression and inflammatory responses, including angiogenesis, epithelial–mesenchymal transition, IL6–JAK–STAT3 signaling, E2F targets, and allograft rejection, exhibited lower GSVA scores in *CAMK2A*-high gliomas (Figure 6B).

To determine whether the relationship between *CAMK2A* and KRAS signaling was independent of clinicopathological variables, multivariable linear regression analysis was performed. *CAMK2A* expression remained significantly associated with the HALLMARK_KRAS_SIGNALING_DN score after adjustment for WHO grade, age, IDH mutation status, MGMT promoter methylation status, chemotherapy, radiotherapy, and 1p/19q codeletion status (Figure 6C). *CAMK2A* was positively associated with HALLMARK_KRAS_SIGNALING_DN (Figure 6C), while analysis of the direct HALLMARK_KRAS_SIGNALING_UP signature showed a consistent inverse trend (Supplementary Figure S2), suggesting a possible inverse relationship between *CAMK2A* expression and KRAS-associated transcriptional activity.

We next evaluated the relationship between *CAMK2A* expression and tumor microenvironment-related features. *CAMK2A* expression was negatively correlated with ESTIMATE, immune, and stromal scores in both the TCGA-GBMLGG and TCGA-LGG cohorts, whereas no significant correlations were observed in the TCGA-GBM cohort (Figure 6D).

To further define the cellular states associated with *CAMK2A* expression, ssGSEA-based lineage scoring was performed using the manually defined cell-type-like marker sets described in Section 2.8. After adjustment for non-tumor proxy, WHO grade, age, and IDH mutation status, *CAMK2A* expression showed the strongest positive association with neuron-like scores and was also positively associated with astrocyte-like and mature oligodendrocyte-like scores (Figure 6E). In contrast, no significant associations were detected with OPC-like or pericyte-like signatures.

### 3.6. Single-Cell Analyses Identify Preferential CAMK2A Expression in Neuronal Populations

**Figure 7:**
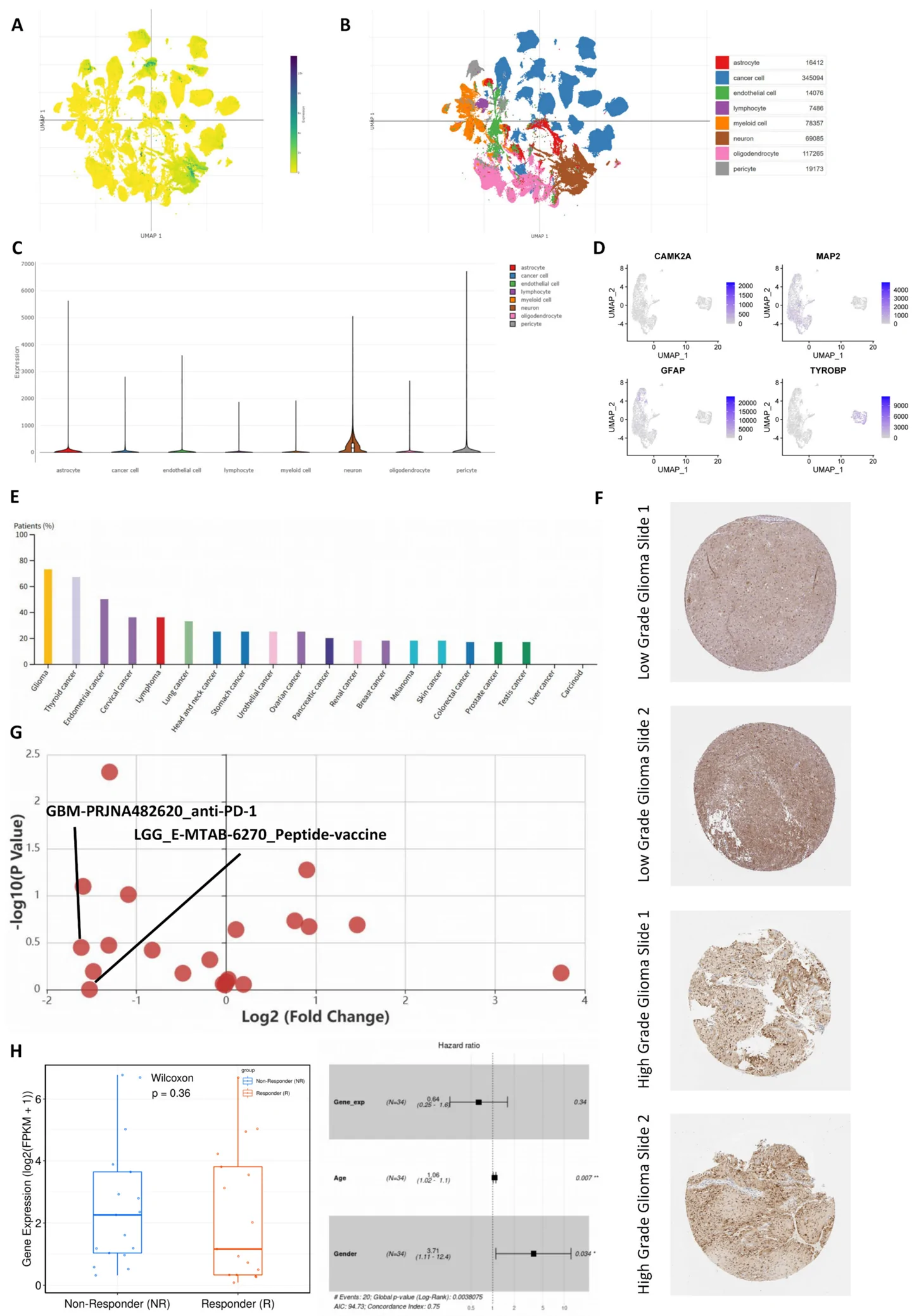
Single-cell transcriptomic characterization and clinical relevance of *CAMK2A* in glioma. (**A**) UMAP visualization of *CAMK2A* expression in glioma single-cell RNA-sequencing data. (**B**) Cell-type annotation of the single-cell dataset. (**C**) Distribution of *CAMK2A* expression across major cell populations. (**D**) UMAP visualization of *CAMK2A, MAP2, GFAP,* and *TYROBP* expression. (**E**) Distribution of *CAMK2A* expression across Human Protein Atlas cancer cell lines. (**F**) Representative immunohistochemical staining of CaMKII in low-grade and high-grade glioma tissues obtained from the Human Protein Atlas. (**G**) Differential expression of *CAMK2A* in published immunotherapy cohorts. (**H**) *CAMK2A* expression in anti-PD-1 responders and non-responders with GBM and Cox regression analysis of overall survival among treated patients. * *p* < 0.05 and ** *p* < 0.01.

Single-cell RNA-sequencing datasets were analyzed to characterize the cellular distribution of *CAMK2A* in glioma. UMAP clustering identified major cellular populations, including cancer cells, neurons, astrocytes, oligodendrocytes, endothelial cells, myeloid cells, lymphocytes, and pericytes (Figure 7A,B). Consistent with the UMAP analysis, violin plot analysis revealed a neuron-enriched expression pattern of *CAMK2A*, with neuronal cells exhibiting the highest expression levels among the annotated cellular populations, whereas only low expression levels were detected in most other cell types (Figure 7C).

To further define the cellular localization of *CAMK2A*, its expression pattern was compared with representative markers of neuronal, astrocytic, and myeloid lineages. *CAMK2A* showed a distribution pattern most similar to that of the neuronal marker *MAP2*, whereas *GFAP* and *TYROBP* were predominantly enriched in astrocytic and myeloid cell compartments, respectively (Figure 7D). These findings support a neuron-associated expression pattern, but similarity to *MAP2* expression alone does not establish malignant-cell identity.

Analysis of Human Protein Atlas data demonstrated detectable *CAMK2A* protein expression across multiple human cancer types (Figure 7E). Consistently, immunohistochemical staining confirmed *CAMK2A* protein expression in both low-grade and high-grade glioma tissues (Figure 7F).

To explore the potential relationship between *CAMK2A* and immunotherapy response, publicly available treatment cohorts were screened, including the anti-PD-1-treated GBM cohort PRJNA482620 and the LGG peptide vaccine cohort E-MTAB-6270 (Figure 7G). In the anti-PD-1-treated GBM cohort, *CAMK2A* expression did not differ significantly between responders and non-responders. Separately, the platform-derived Cox analysis did not identify a significant association between *CAMK2A* expression and overall survival among treated patients (Figure 7H). Thus, these exploratory analyses do not establish *CAMK2A* as a marker of anti-PD-1 response in GBM.

### 3.7. Progressive Loss of CaMKII and MAP2 Expression During Glioma Progression

**Figure 8:**
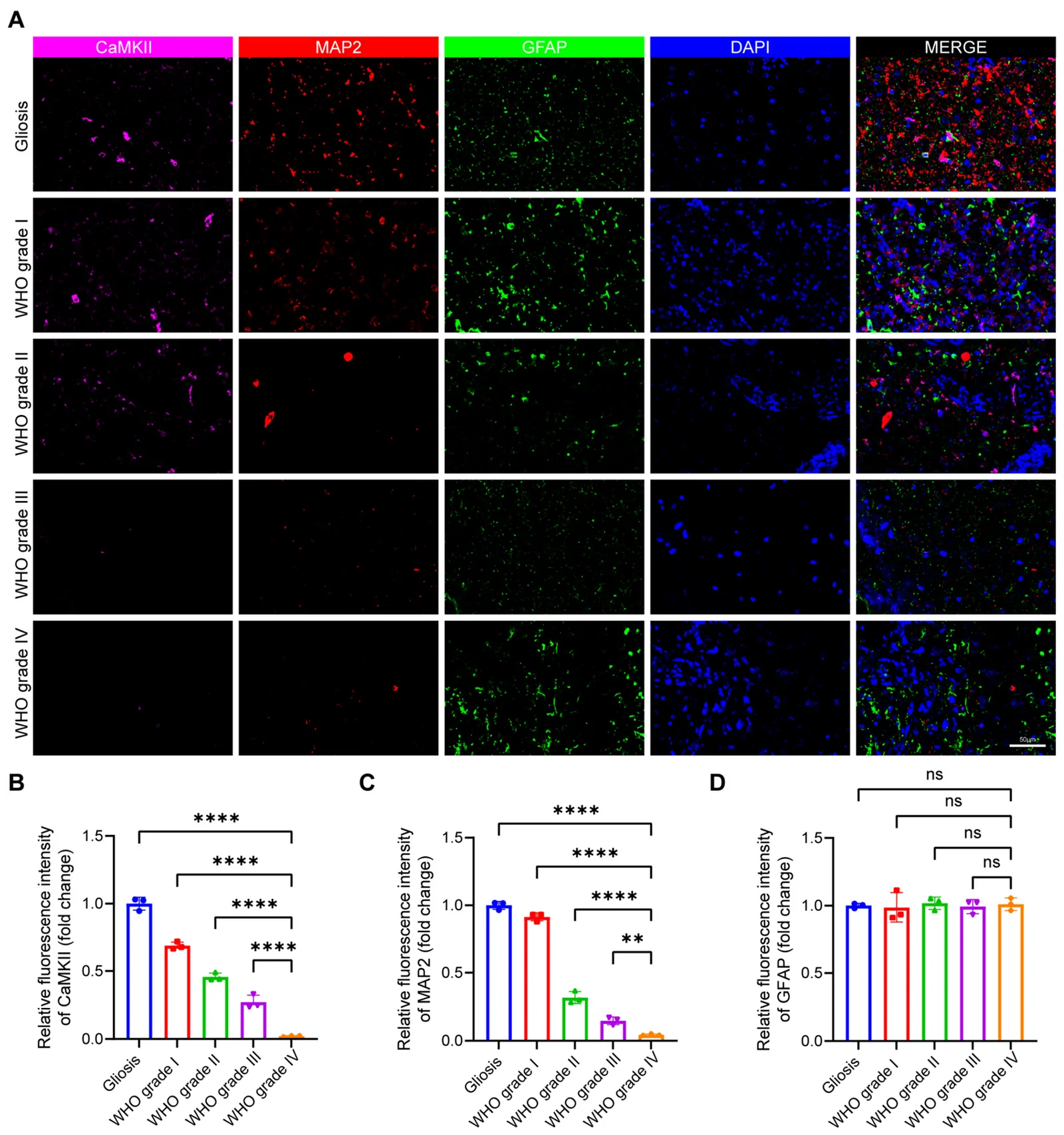
Multiplex immunofluorescence validation of CaMKII, MAP2, and GFAP expression in glioma tissues. (**A**) Representative multiplex immunofluorescence staining of CaMKII (magenta), MAP2 (red), GFAP (green), and DAPI (blue) in gliosis tissues and WHO grade I–IV glioma specimens. Scale bar = 50 μm. (**B**–**D**) Quantification of the relative fluorescence intensities of CaMKII (**B**), MAP2 (**C**), and GFAP (**D**). Data are presented as mean ± SEM. Statistical significance was determined by one-way ANOVA followed by Dunnett’s multiple comparisons test. ** *p* < 0.01; **** *p* < 0.0001; ns, not significant.

To validate the neuron-associated molecular features identified by the *CAMK2A* transcriptomic analyses at the tissue level, multiplex immunofluorescence staining for CaMKII, MAP2, GFAP, and DAPI was performed in gliosis tissues and glioma specimens spanning WHO grades I–IV (Figure 8A). Strong CaMKII and MAP2 signals were detected in gliosis tissues and WHO grade I gliomas. As tumor grade increased, the staining intensities of both markers progressively declined, with only sparse positive signals observed in WHO grade III and grade IV tumors (Figure 8A).

Quantitative analyses confirmed a grade-dependent reduction in CaMKII expression, with significantly lower fluorescence intensities detected in WHO grade II–IV gliomas compared with gliosis tissues and lower-grade gliomas (Figure 8B). MAP2 exhibited a similar expression pattern, showing a progressive decline across increasing WHO grades and reaching its lowest level in grade IV tumors (Figure 8C). In contrast, GFAP fluorescence intensity remained relatively stable across gliosis tissues and gliomas of different grades, with no significant differences observed among groups (Figure 8D).

Taken together, these results demonstrate a coordinated loss of CaMKII and MAP2 during glioma progression, in agreement with the transcriptomic and single-cell analyses showing reduced CaMKII expression and diminished neuron-associated features in high-grade gliomas.

## 4. Discussion

Growing evidence indicates that neuronal differentiation programs represent an important dimension of glioma heterogeneity [19,93,94,95]. In the present study, integrated analyses of bulk transcriptomic datasets, single-cell sequencing profiles and clinical tissue specimens identified *CAMK2A* as a neuron-associated molecular marker whose expression gradually declines as glioma malignancy advances, and high *CAMK2A* expression correlates with favorable patient survival. More importantly, consistent evidence across multiple analytical platforms links *CAMK2A* abundance to neuron-like transcriptional signatures, revealing an underrecognized connection between neuronal differentiation status and glioma aggressiveness. Collectively, these results support *CAMK2A* as a candidate prognostic biomarker associated with neuronal transcriptional features in glioma specimens; the relative contributions of malignant cells and residual normal neurons remain to be resolved.

A core finding of this work is the tight correlation between *CAMK2A* expression and neuronal transcriptional programs. Functional enrichment analyses via GO and KEGG pathways uniformly showed that *CAMK2A*-coexpressed genes are mainly involved in synaptic assembly, neurotransmitter transport, calcium signal transduction and neuronal projection morphogenesis. Tissue-level immunofluorescence validation further confirmed coordinated expression of *CAMK2A* and MAP2, a canonical marker of mature neuronal differentiation. At single-cell resolution, *CAMK2A* transcripts were preferentially enriched in populations annotated as neurons, with low expression in most other populations (Figure 7A–C). These data localize the neuronal signal but do not, by themselves, demonstrate a tumor-intrinsic neuron-like state.

An important alternative explanation is normal neuronal admixture in bulk RNA-sequencing samples. Residual neurons entrapped within or adjacent to lower-grade tumors could contribute both *CAMK2A* transcripts and neuronal signature genes, generating their correlation without an intrinsic neuron-like program in glioma cells. The stromal and immune proxy adjustment used here does not specifically quantify or remove normal neuronal contributions, and tissue-level CaMKII/MAP2 staining likewise cannot establish malignant identity. Although the single-cell observations are compatible with neuron-associated biology, enrichment in an annotated neuronal population does not distinguish normal neurons from neuron-like malignant cells without independent genetic evidence. Future analyses should identify malignant cells using copy-number alterations and/or tumor-specific mutations, address ambient RNA and doublets, and then test *CAMK2A*-signature associations within the malignant compartment. Spatial RNA or protein measurements combined with tumor-specific genetic markers would provide complementary validation. Accordingly, the present findings support a neuron-associated signal in glioma tissue but do not exclude normal neuronal contamination as a contributor, especially in lower-grade specimens.

This molecular linkage is biologically interpretable given the well-characterized physiological function of *CAMK2A* in the central nervous system. *CAMK2A* is one of the most abundant serine/threonine kinases in excitatory neurons and acts as a central effector of calcium-triggered neuronal signaling, synaptic plasticity, learning and memory [1,2]. As an indispensable constituent of postsynaptic signaling complexes, *CAMK2A* is widely regarded as a marker of mature excitatory neuronal differentiation [9]. Accordingly, the enrichment of neuronal and synaptic pathways observed in *CAMK2A*-high gliomas may reflect neuronal programs within malignant cells, residual normal neurons, or both.

Consistent with prior single-cell studies demonstrating dynamic cell-state heterogeneity in gliomas [19], neuron-like tumor subpopulations have been recognized as a critical component driving tumor progression. Multiple studies have demonstrated that subsets of glioma cells activate neuronal lineage and differentiation programs and exhibit transcriptional features reminiscent of normal neural development [95,96], and neuronal activity can remodel tumor microenvironments via neuron-glioma synaptic crosstalk [97,98,99]. Building on these foundational findings and the emerging framework of cancer neuroscience [100], our data identify *CAMK2A* as a neuron-associated marker in glioma specimens, while its specificity for neuron-like malignant cells remains unproven.

Importantly, our finding that high *CAMK2A* expression is associated with lower tumor grade, favorable prognosis, and a more differentiated phenotype suggests that *CAMK2A*-associated neuronal features should not be equated directly with malignant neuron–glioma synaptic integration. Venkatesh et al. demonstrated that functional AMPA receptor-dependent neuron–glioma synapses and activity-induced depolarization promote the growth of high-grade gliomas, with synapse-related transcriptional programs enriched in OPC-like malignant cells [18]. Similarly, Venkataramani et al. showed that, within glioblastoma, neuronal- and neural-progenitor-like tumor cells receiving neuronal synaptic input constitute an invasive population, in which neuronal activity induces calcium signaling, tumor-microtube formation, and increased invasion [99]. These findings indicate that neuronal-like molecular features can coexist with aggressive, synaptically driven tumor behavior, but do not imply that all neuronal differentiation programs confer such a phenotype. In this context, *CAMK2A*-high gliomas in our study may represent a more differentiated neuronal-like state that is biologically distinct from the synaptically integrated invasive states described in high-grade glioma and glioblastoma. Thus, *CAMK2A* expression alone should not be considered evidence of increased functional neuron–glioma synaptic activity. From a therapeutic perspective, disruption of neuron–glioma communication, particularly AMPA receptor-dependent signaling, remains a potential strategy in synaptically integrated gliomas [100]. However, our findings do not support *CAMK2A* itself as a therapeutic target and instead suggest that it may serve as a marker of tumor differentiation state. Direct electrophysiological and spatial studies with independent confirmation of malignant-cell identity will be required to determine whether *CAMK2A*-expressing glioma cells participate in neuron–glioma circuitry.

The lower *CAMK2A* expression observed at higher glioma grades motivates a testable hypothesis of gradual erosion of neuronal lineage-associated transcriptional features. Under this hypothesis, some malignant cells may retain neuronal programs, and reduced *CAMK2A* expression may accompany a shift away from these programs. However, our data are cross-sectional and correlative: comparisons across grades and molecular subtypes do not reconstruct the evolution of an individual tumor, establish that *CAMK2A* loss causes dedifferentiation, or imply that normal mature neurons are glioma precursors. This model is therefore a conceptual hypothesis rather than a demonstrated sequence of malignant progression. Differences in cellular composition provide an additional explanation that must be separated from changes within malignant cells.

Prior studies provide a framework for considering glioma cell-state plasticity, but do not establish the specific *CAMK2A*-dependent trajectory proposed here. Neftel et al. demonstrated bidirectional cell-state switching in GBM [19]; developmental hierarchy models highlight the strong lineage plasticity of glioma cells [93]; additional work further confirms microenvironment-induced state transition and links dedifferentiation to therapeutic resistance [101,102]. In accordance with these frameworks, our tissue cohort analyses detected synchronous downregulation of *CAMK2A* and *MAP2* as pathological grade increases. Collectively, these observations are compatible with the proposed hypothesis, but longitudinal sampling and lineage-resolved experiments are needed to distinguish state transitions from differences between tumors.

This hypothesis could be tested by CRISPR-mediated *CAMK2A* knockout in patient-derived glioma cultures with detectable endogenous expression and by inducible *CAMK2A* overexpression in low-expressing cultures, using multiple molecular backgrounds, independent guide RNAs, non-targeting controls, and rescue with an editing-resistant *CAMK2A* construct. Neuronal differentiation should be assessed using *MAP2, SYP, NRN1, RBFOX3* and broader transcriptional programs, alongside EdU incorporation, Ki-67, cell counts and viability to distinguish changes in differentiation from selective cell loss or altered proliferation. A time course with single-cell profiling would help resolve state changes within malignant cells. Rescue experiments, including comparison with a kinase-inactive construct, could test whether any observed effects depend on *CAMK2A* kinase activity. These experiments have not been performed in the present study.

*CAMK2A* should also be evaluated in the context of broader neuronal signatures. The neuron-like score used here comprises *RBFOX3, SNAP25* and *SYT1* and excludes *CAMK2A* (Section 2.8); its association with *CAMK2A* therefore provides evidence of concordance with a neuronal program, but does not establish superiority over individual neuronal markers. We did not perform a head-to-head comparison with *SYP, NRN1* or *RBFOX3*, or benchmark *CAMK2A* against the published Neftel cell-state modules. The latter describe NPC-like, OPC-like, AC-like and MES-like malignant states and should not be equated with a mature-neuronal signature. A suitable comparison would evaluate *CAMK2A* and the alternative markers against independently defined neuronal programs and published state scores within genetically supported malignant cells, using patient-level held-out validation. Evaluated genes should be excluded from the reference score to avoid circularity, and bulk analyses should account for grade, IDH status, tumor purity and neuronal admixture. Testing whether adding *CAMK2A* improves performance beyond a broader signature would establish its incremental value. Until such benchmarking is available, *CAMK2A* is best regarded as a candidate readout of a broader neuronal program; its uniqueness and relative predictive performance remain unresolved.

We also detected an inverse correlation between *CAMK2A* expression and multiple oncogenic signaling axes linked to malignant phenotypes. Tumors with abundant *CAMK2A* displayed attenuated activity of mesenchymal transition, pro-inflammatory signaling and angiogenesis pathways, all canonical hallmarks of aggressive glioma behavior. Notably, mesenchymal transformation represents the most malignant cellular substate in glioblastoma and closely correlates with therapeutic resistance and dismal clinical outcomes [19,103]. Chronic inflammatory activation and neovascularization are also essential drivers of glioblastoma progression and microenvironment remodeling [104,105]. The negative association between *CAMK2A* and these malignant pathways therefore indicates that *CAMK2A*-high tumors correspond to a relatively differentiated, less aggressive cellular phenotype. At present, it remains unclear whether *CAMK2A* directly represses these oncogenic pathways or merely passively mirrors retained neuronal differentiation; dedicated functional experiments are required to resolve this question.

From a translational clinical perspective, our data validate the prognostic utility of *CAMK2A* in diffuse gliomas. Across several independent patient cohorts, elevated *CAMK2A* expression consistently predicted prolonged overall survival, and this prognostic value remained statistically significant after adjustment for established molecular biomarkers such as IDH mutation status. Given the profound biological heterogeneity inherent to diffuse gliomas [106], biomarkers reflecting tumor cell differentiation status can deliver complementary prognostic information beyond conventional clinicopathological parameters. Nevertheless, the current correlative evidence does not qualify *CAMK2A* as an immediate actionable therapeutic target. Instead, our findings support its application as a molecular tool for glioma biological subtyping, risk stratification and future development of differentiation-targeted therapeutic strategies.

The immunotherapy results define an important boundary condition for the proposed biomarker utility of *CAMK2A*. The absence of a significant association with anti-PD-1 response in GBM (Figure 7G,H) indicates that the favorable prognostic association observed across glioma cohorts cannot be assumed to predict immunotherapy benefit. Low *CAMK2A* expression in high-grade GBM offers a plausible explanation: low transcript abundance could reduce the sensitivity of expression-based stratification or reflect diminished representation of *CAMK2A*-associated neuronal programs. It is also conceivable that low expression limits a putative immunomodulatory effect, but neither a direct immunomodulatory role nor a functional expression threshold was tested here. We therefore cannot attribute the lack of association to this mechanism. Clinical heterogeneity and limited statistical power are alternative explanations, and nonsignificance does not establish biological irrelevance. The current evidence does not support *CAMK2A* alone for selecting patients for anti-PD-1 therapy. Larger, independently validated treatment cohorts, ideally including a comparator arm and a *CAMK2A*-by-treatment interaction analysis, are needed to distinguish prognostic associations from prediction of treatment benefit.

Several limitations of this study must be acknowledged. First, most transcriptomic analyses rely on publicly available datasets, which may introduce cohort-specific bias despite cross-cohort validation. Second, although single-cell and tissue-level observations link *CAMK2A* to neuronal features in glioma specimens, our observational analyses cannot establish causal regulatory relationships. Third, loss-of-function and gain-of-function functional assays were not performed; hence the direct biological function of *CAMK2A* in glioma progression remains uncharacterized. Fourth, all single-cell datasets analyzed were retrieved from published cohorts and may not fully capture the full spectrum of neuron-like substates across all glioma molecular subtypes. Finally, the detailed molecular cascades connecting *CAMK2A* to neuronal differentiation, cellular plasticity and malignant progression require rigorous in vitro and in vivo mechanistic validation.

## 5. Conclusions

In summary, this study identifies *CAMK2A* as a neuron-specific marker progressively downregulated during glioma progression, with high expression independently predicting superior clinical outcomes. *CAMK2A* abundance correlates with neuronal transcriptional signatures in glioma specimens, whose malignant-cell and normal-neuronal contributions remain incompletely resolved, while showing inverse association with multiple oncogenic pathways. These findings uncover an undervalued linkage between neuronal differentiation degree and glioma malignancy, offering novel insights into the molecular basis of glioma inter-tumor and intra-tumor heterogeneity. *CAMK2A* therefore may serve as a biomarker for prognostic evaluation and biological stratification of patients with diffuse gliomas. Future functional in vitro and in vivo assays are required to clarify the causal relationship between *CAMK2A* and neuronal differentiation, while combined immunohistochemical detection of *CAMK2A* and *MAP2* may provide a convenient clinical panel for glioma risk stratification.

## Data Availability

The datasets analyzed in this study are publicly available from TCGA, CGGA, GTEx, CPTAC, and other open-access repositories. The corresponding accession numbers and sources are provided in Section 2. Additional data generated during this study are available from the corresponding author upon reasonable request.

## Supplementary Materials

The following supporting information can be downloaded at: https://www.mdpi.com/article/10.3390/biomedicines14102216/s1. Figure S1: Sensitivity analysis of the association between CAMK2A expression and overall survival in glioma using median expression cutoffs.; Figure S2: Multivariable analysis of the association between CAMK2A expression and the Hallmark KRAS signaling UP signature in glioma.
